# Perceived neighborhood built environment and its associations with sociodemographic characteristics among older adults in regional China

**DOI:** 10.3389/fpubh.2026.1909471

**Published:** 2026-09-07

**Authors:** Yu Dou, Hongyun Zhou, Qing Ye, Zhiyong Wang, Caihong Hu, Guofeng Ao, Luyan Yang, Yaqing Xiong, Fei Xu

**Affiliations:** 1 Jiangsu Province Official Hospital, Nanjing, China; 2 Geriatric Hospital of Nanjing Medical University, Nanjing, China; 3 Nanjing Medical University Affiliated Nanjing Municipal Center for Disease Control and Prevention, Nanjing, China; 4 Nanjing Medical University School of Public Health, Nanjing, China

**Keywords:** built environment, Chinese, older adults, perception, physical activity, socio-demographic characteristics

## Abstract

**Background:**

Neighborhood built environment (BE) is associated with physical activity (PA) and health-related conditions among local residents, particularly older adults. As an easily operational instrument, the Physical Activity Neighborhood Environment Scale (PANES) has been widely used to measure BE attributes related to PA globally. However, evidence remains limited on how older adults with different sociodemographic characteristics perceive local BE attributes in China. This study aimed to investigate the associations between sociodemographic characteristics and BE attributes among older residents in regional China.

**Methods:**

In this cross-sectional survey conducted in Nanjing Municipality, China, in 2023, 5,484 community-dwelling adults aged 60 + years were randomly selected. Six out of seven objectively measurable PANES core items were used to assess BE attributes, and they were treated as outcome variables. Item 1 recorded the exact housing type, while each of the remaining five items was classified as “strongly/somewhat disagree” or “strongly/somewhat agree” based on a 4-point Likert scale. The explanatory variables were the classical sociodemographic characteristics. Mixed-effects multivariate logistic regression models were used to investigate the associations between sociodemographic characteristics and BE attributes.

**Results:**

After adjusting for potential confounders, suburban residents were less likely to perceive PA-favorable commercial facilities, public transportation transit/stops, sidewalks on streets, shared paths for cycles and pedestrians, and free/low-cost recreational facilities. Blue-collar workers were less likely to perceive PA-supportive sidewalks on streets and shared paths for cyclists and pedestrians. Participants with middle-level educational attainment were more likely to report PA-friendly commercial facilities, sidewalks, shared paths for cycles and pedestrians, and free/low-cost recreational facilities, whereas individuals with a spouse/partner tended to report sidewalks and shared paths for cycles and pedestrians. Interestingly, participants aged 70–79 years were less likely to live in a single house, and no link was observed between sex and perceived BE attributes.

**Conclusion:**

Residential location, education, occupation, and marital status were all associated with perceived BE attributes among older residents in regional China; however, age was only associated with the type of housing. This study has important public health implications, suggesting that sociodemographic characteristics may serve as helpful markers for screening older community-dwelling adults who may perceive PA-supportive BE attributes in regional China.

## 1 Introduction

The lack of physical activity (PA) has become a global public health concern, highlighting the need for community-level strategies to promote PA and reduce sedentary behavior (SB) (1). However, it is a significant challenge to help residents sustainably promote and maintain PA at the population level. Fortunately, recent research shows that the neighborhood built environment (BE) is associated with PA and health-related chronic conditions (2–4). In particular, some BE attributes such as residential density, land use, and accessibility to public transport systems have been identified as PA-favorable in population studies (5–7). As the built environment, once established, can significantly influence the behaviors of individuals who reside in the neighborhoods, it is of public health importance to further investigate the relationship between BE attributes and PA, particularly the potential factors that may affect the utilization of a PA-friendly BE.

Theoretically, residents can utilize PA-friendly BE only when they are aware of its availability and potential benefits. This conceptual framework is underpinned by the Knowledge–Attitude–Practice (KAP) model (8). The KAP theory posits that knowledge is the primary basis for subsequently modifying behaviors/practices by establishing a positive attitude toward health-related consequences (8). It emphasizes that knowledge or awareness is the first step toward behavioral change (8). Therefore, according to the KAP model, to promote PA through favorable neighborhood BE utilization, the logical operational framework involves improving local residents’ awareness and perceptions of PA-friendly BE attributes and strengthening their positive attitudes toward PA-related health outcomes.

Typically, neighborhood BE can be assessed using objective methods such as geographic information systems (GISs) (9) or subjective approaches such as the Physical Activity Neighborhood Environment Scale (PANES) (10). Usually, when residents self-report their perceptions of BE attributes, it indicates that they are aware of the neighborhood’s BE and may subsequently utilize its favorable attributes. Previous studies have also demonstrated that individuals’ health may be more strongly affected by their self-perceived neighborhood BE attributes than by objectively measured neighborhood BE attributes (11, 12). Moreover, the association between objectively measured neighborhood BE attributes and health conditions may be mediated by self-perceived attributes (12, 13). Therefore, identifying the factors associated with the perception of BE is necessary to inform the potential utilization of favorable neighborhood BE attributes.

Sociodemographic characteristics typically reflect residents’ social position and have been shown to be associated with awareness/knowledge of health-related behaviors and/or their consequences (14–17). Moreover, sociodemographic characteristics have been reported to be moderators of the relationship between perceived BE attributes and PA (18–20). Additionally, it has also been documented that participants from different sociodemographic subgroups may report BE attributes differently in mainland China, Hong Kong, and Korea (21–23). However, among the six classical sociodemographic characteristics (age, sex, residential area, education, occupation, and marital status) and the four main domains of health-friendly BE attributes (residential density, land-use mix, access to destinations, and neighborhood infrastructure), only a few have been investigated separately in existing studies (21–23). No study has simultaneously examined the associations between the classical sociodemographic characteristics and the four domains of BE. Therefore, from a public health perspective, it is meaningful to explore the associations between the classical sociodemographic characteristics and perceived BE attributes among community-dwelling adults, as this may help residents make better use of PA-friendly BE attributes (24).

The PANES, an independent environmental module of the International Prevalence Study on Physical Activity (IPS), is a widely used instrument to measure neighborhood BE attributes via self-perception (9). In addition to the easiness of operation, the reliability and validity of the PANES have been examined across different languages and countries/territories, including China (25–30). However, to date, the association between PANES-measured neighborhood BE attributes and sociodemographic characteristics has not been investigated among residents, particularly older adults, in China. To address this gap, we hypothesized that sociodemographic characteristics may be associated with perceived BE attributes among older adults. To test this hypothesis, we examined the relationship between PANES-assessed PA-related BE attributes and the six typical sociodemographic characteristics among community-dwelling older adults in regional China.

## 2 Methods

### 2.1 Study design and participants

Data analyzed in this cross-sectional study were derived from the Healthy Aging and Healthy Older Residents study (HAHOR study), which were gathered from community-dwelling older adults in Nanjing Municipality of China between early July and early December 2023 (31–33). In total, Nanjing had 12 administrative districts and approximately 9.3 million registered inhabitants in 2020 (34). Moreover, among the overall local population, 20.9% were adults aged 60 years or above (34). The participants and sampling procedures have been described in detail elsewhere (31–33). Briefly, the inclusion criteria were as follows: (1) being a local community-dwelling registered inhabitant in Nanjing; (2) aged 60 years or above; (3) having no linguistic, cognitive, or mental disorders; (4) having no restrictions in daily activities; and (5) not having an acute infectious disease (31–33).

The sample size for the HAHOR study was estimated considering the following factors: (1) the nature of the population-based cross-sectional survey; (2) the reported prevalence of the primary outcome event, fatigability/fatigue (27.0%), among community-dwelling adults aged 60 years and older (35, 36); (3) the multistage sampling approach employed; (4) a statistical power of 90%; (5) an assumed response rate of 90%; and (6) the stratified data analysis method (31–33). Finally, it was determined that the overall sample size should be approximately 6,000 for the HAHOR study, which was also sufficient for examining the association between sociodemographic characteristics and BE attributes in the present study (14, 15, 20).

**Figure 1:**
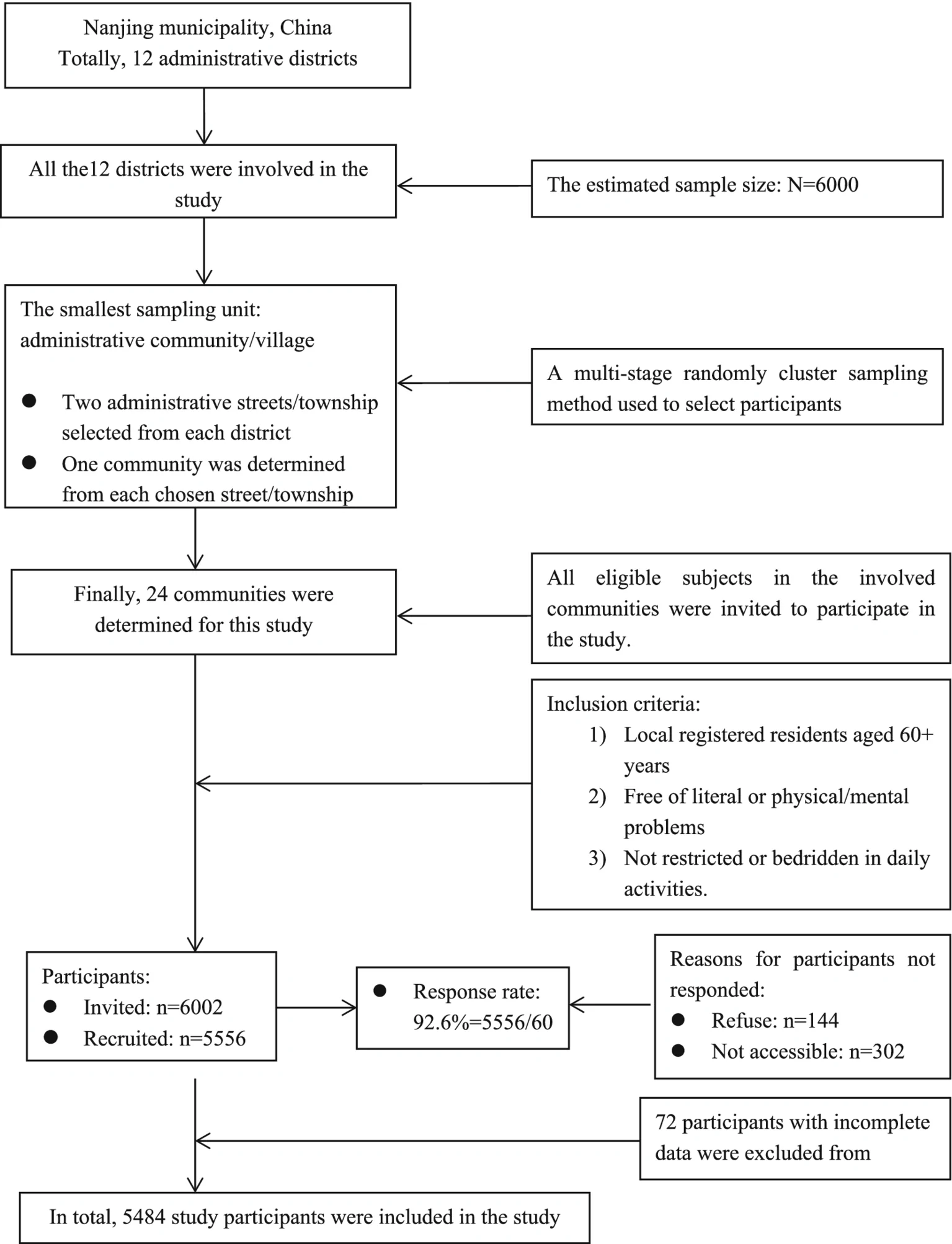
Flowchart of participant selection in this study.

For the recruitment of participants, a multistage sampling method was employed to randomly select participants from all 12 districts of Nanjing (31–33). First, based on the five-level administrative system (central government, province/municipality, district/county, streets/townships, and residential community) in China, two streets/townships were randomly selected from each district in Nanjing. Second, one residential community was selected from each determined street/township. Third, considering the number of sample size (*N* = 6,000) estimated and the total communities (*N* = 24) involved, 250 eligible participants were recruited from each selected residential community. Therefore, based on the residential community household list, 250 eligible participants were determined from each community (31–33) (Figure 1)

Written informed consent was obtained from all participants before the survey. This study was reviewed and approved by the ethics committee of the Nanjing Medical University Affiliated Nanjing Municipal Center for Disease Control and Prevention. The study protocol and experiment were developed in line with the recommended principles of the Declaration of Helsinki.

### 2.2 Data collection

Information collected from participants mainly included neighborhood BE attributes, sociodemographic and anthropometric characteristics, lifestyle and behavioral factors, and selected non-communicable diseases (NCDs), using a standardized questionnaire (31–33). All these data were measured using instruments and definitions recommended in the “Scheme of the Chinese Chronic Non-communicable Diseases and Risk Factor Surveillance” by the Chinese Center for Non-communicable Disease Control and Prevention (CCNDC) (37). Neighborhood BE attributes, such as PA, SB, sleep, frailty, and depressive symptoms, were assessed using specific validated scales (9, 37–41). Moreover, the field survey procedures and quality control approaches were also adopted from the *scheme* issued by the CCNDC (37).

### 2.3 Study variables

#### 2.3.1 Outcome variable

The outcome measures were perceived neighborhood BE attributes, assessed using the core items of the PANES (10). A neighborhood was defined as a residence area within a 10–15 min walking distance of the participant’s home, corresponding to approximately 1.0 km (42). The PANES includes 17 items, categorized as “core” (seven items), “recommended” (four items), and “optional” (six items) (10). The core items are compulsorily required to be used to describe BE attributes in population-based studies examining the BE and health, while the recommended and optional items are discretionary (10). The core items capture all the key domains of PA-related BE attributes—residential density (item 1), land-use mix (item 2), access to destinations (item 3), neighborhood infrastructure (items 4, 5 and 6), and neighborhood safety (item 7)—and thus are widely used to measure BE attributes alone (10). The seven core items of the PANES are as follows: Item 1, “What is the main type of housing in your neighborhood?”; item 2, “Many shops, stores, markets, or other places to buy things I need are within easy walking distance of my home”; item 3, “It is within a 10–15-min walk to a transit stop from my home”; item 4, “There are sidewalks on most of the streets in my neighborhood”; item 5, “There are facilities to bicycle in or near my neighborhood, including shared-use paths for cycles and pedestrians”; item 6, “My neighborhood has several free or low-cost recreation facilities”; and item 7, “The crime rate in my neighborhood makes it unsafe to go on walks at night” (10).

In the present study, the seven core items were used to assess self-perceived neighborhood BE attributes. Given that perceived physical neighborhood BE attributes may contribute more than perceived social features to health conditions and their associated behaviors (12), item 7, which describes neighborhood attributes of social safety rather than the physical aspects of built environment, was excluded from the analysis. For the remaining six objectively measurable items, item 1 assessed the exact housing type, while the other five items used a 4-point Likert scale (1 = strongly disagree, 2 = somewhat disagree, 3 = somewhat agree, and 4 = strongly agree) (10). In previous studies examining the validity and reliability of the PANES, responses to all items using the four-point Likert scale were classified as binary measures (10, 29, 30). Therefore, to warrant comparability, answer options were also dichotomized in this study. Responses to item 1 were categorized as “0” (apartment) or “1” (house) in the context of China, while answers to items 2–6 were classified as “0” (strongly/somewhat disagree) or “1” (strongly/somewhat agree) (10, 29, 30). Clearly, a PA-unfavorable BE attribute was coded as “0,” while a PA-friendly BE attribute was coded as “1” for each item in the analysis.

#### 2.3.2 Explanatory variables

The explanatory variables comprised typical sociodemographic characteristics, including age, sex, residential area, educational attainment, marital status, and occupation (31–33). The classifications of these six common sociodemographic characteristics were as follows: Age (60–69 years [younger-old], 70–79 years [middle-old], or ≥80 years [oldest-old]), sex (male or female), residential location (urban or suburban), education (≤6, 7–12, or ≥13 years of schooling), marital status (single or having a partner/spouse), and pre-retirement occupation (white-collar, service, or blue-collar occupations) (31–33).

#### 2.3.3 Covariates

This study aimed to investigate the relationship between perceptions of BE attributes and sociodemographic characteristics. It has been documented that local residents who are physically inactive tend to be less aware of or perceive less favorably the neighborhood built environment (43). Therefore, PA and its associated factors were treated as covariates in multivariate analyses, including PA, SB, selected NCDs, sleep, frailty, depressive symptoms, and body weight status. PA and SB were assessed using the validated Chinese version of the International Physical Activity Questionnaire (IPAQ-CHN) (38). Participants self-reported their PA frequency, intensity, and duration in the past 7 days. Then, the total weekly moderate-to-vigorous PA (MVPA) time was computed as the sum of moderate PA time and twice the vigorous PA time during the previous week. This was then used to classify participants as having “insufficient PA (<150 min/week)” or “sufficient PA (≥150 min/week)” (44). SB was assessed based on daily screen-viewing time and used to categorize participants as having “prolonged SB (≥2 h/day)” or “shortened SB (<2 h/day),” according to the cutoffs specifically recommended for Chinese adults (44).

Selected NCDs included self-reported diabetes, hypertension, chronic obstructive pulmonary disease (COPD), coronary heart disease (CHD), stroke, chronic kidney disease (CKD), cancer, and abnormal lipid profiles. Diabetes, hypertension, COPD, CHD, CKD, and cancer were each recorded as “Yes” or “No” responses based on the information self-reported by participants. Participants were classified as having a “normal lipid profile” only if their self-reported cholesterol, triglyceride, and high- and low-density lipoprotein levels were all within the normal ranges; otherwise, they were categorized as having an “abnormal lipid profile” (31–33). Sleep quality was assessed using the Pittsburgh Sleep Quality Index (PSQI), with a cutoff value of 5 used to classify participants as having “poor (≥5)” or “good (<5)” sleep quality (45). Frailty was assessed using the Groningen Frailty Indicator (GFI), with a cutoff score of 3 used to categorize participants as “frail (GFI ≥ 3)” or “not frail (GFI < 3)” (40). Depressive symptoms were assessed using the Patient Health Questionnaire-9 (PHQ-9), with a score of 5 used to determine participants as “having (≥5)” or “not having (<5)” depressive symptoms (41).

Body weight and height were objectively measured based on the procedures suggested by the CCNDC (37). Briefly, participants (barefoot and with light clothing) had their weight and height measured twice, with measurements recorded to the nearest 0.1 kg and 0.01 meter, respectively. Then, the mean values were used to compute body mass index (BMI), which is determined by dividing body weight (kg) by height squared (m^2^) (37). Based on the BMI cutoffs recommended for Chinese adults, participants were categorized into “normal weight (BMI<24.0),” “overweight (24.0 ≤ BMI < 28.0),” or “obesity (BMI ≥ 28.0)” (46).

### 2.4 Data analysis

Using the chi-squared test, descriptive analysis was conducted to compare the differences in sociodemographic characteristics by residential location. Next, multivariate mixed-effects logistic regression analyses were conducted to calculate odds ratios (ORs) and 95% confidence intervals (95% CIs) and to examine the associations between sociodemographic characteristics and perceived BE attributes for each item. In the models, all sociodemographic characteristics and potential confounding factor (including PA, SB, body weight status, diabetes, hypertension, stroke, CHD, CKD, COPD, cancer, lipid profiles, sleep quality, frailty, and depressive symptoms) were mutually adjusted for, with the sampling community as the random effect. Data were analyzed using R (version 4.4.3, R Foundation, Vienna, Austria).

## 3 Results

In total, 6,002 eligible participants were randomly selected, and 5,556 were successfully interviewed (response rate = 92.6%). Among the 446 participants who were not recruited, 144 refused to take part in the survey and 302 were not available. Of the 5,556 recruited participants, 72 reported incomplete survey data and were then excluded from the analysis, resulting in a final analytical sample of 5,484 participants. There were no statistical differences in age, sex, or residential area between participants included and not included in the analysis. Table 1 summarizes the selected participants’ characteristics by residential area. Among the participants analyzed, the mean age (standard deviation) was 69.7 (6.8) years, with 57.6, 31.2, and 11.2% aged 60–69, 70–79, and ≥80 years, respectively. Men accounted for 49.0% of the participants, and 39.1% were from urban areas. Moreover, 49.6% had 0–6 years of schooling, while only 5.4% had completed college-level education. Notably, 13.8% of participants did not have a spouse or partner. In addition, the community-level intraclass correlation coefficient and variance were just 0.06 and 1.3, respectively, showing that the community-level clustering effect was very small.

**Table 1:** Selected characteristics of participants by residential area in this study (*N* = 5,484).

Personal characteristics	Total	% (*n*) of participants	*χ* ^2^	*p*-value^*^	Personal characteristics
		Urban ^#^	Suburban ^#^		
Overall	5,484	39.1 (2,144)	60.9 (3,340)		
Age (years)					
60–69	57.6 (3,157)	58.5 (1,255)	56.9 (1,902)		
70–79	31.2 (1,711)	30.4 (650)	31.8 (1,061)	1.48	0.478
80+	11.2 (616)	11.1 (239)	11.3 (377)		
Sex					
Male	49.0 (2,687)	49.4 (1,060)	48.7 (1,627)	0.28	0.599
Female	51.0 (3,797)	50.6 (1,084)	51.3 (1,713)		
Educational attainment (years of schooling)					
0–6	49.6 (2,721)	24.2 (519)	65.9 (2,202)		
7–12	45.0 (2,465)	64.7 (1,387)	32.3 (1,078)	971.40	<0.001
13+	5.4 (298)	11.1 (238)	1.8 (60)		
Marital status					
Single	13.8 (758)	11.6 (248)	15.3 (510)	15.03	<0.001
Married/having a partner	86.2 (4,726)	88.4 (1,896)	84.7 (2,830)		
Occupation ^†^					
White-collar	15.1 (827)	29.8 (638)	5.7 (189)		
Salesperson	6.2 (338)	11.0 (235)	3.1 (103)	803.85	<0.001
Blue-collar	78.7 (4,319)	59.2 (1,271)	91.2 (3,048)		

*Chi-square test.

# Urban and suburban districts were defined based on the official classifications released by the China National Bureau of Statistics.

† Occupation referred to the job before retirement and was classified for each participant based on the recommendations by the China National Center for Chronic Non-communicable Disease Prevention and Control.

Table 2 shows the distribution of the six self-perceived core BE attributes according to participants’ sociodemographic characteristics. Perceived residential density, indicated by housing type (item 1), differed according to residential area, educational attainment, marital status, and occupation. Land-use mix, reflected by either commercial facilities (item 2) or public transportation transit/stops (item 3), also varied across participants according to age, residential area, education, marital status, and occupation. Moreover, self-reported neighborhood infrastructure, indicated by the presence of sidewalks on streets (item 4) and shared paths for cycles and pedestrians (item 5), consistently differed across participants according to age, residential area, education, marital status, and occupation. In contrast, the availability of free/low-cost recreational facilities (item 6) differed across participants according to age, residential area, education, and occupation.

**Table 2:** Seven core items of the PANES for built environment attributes among participants in this study (*N* = 5,484).

		% (*n*) of participants by responses to the core items of the PANES											
Personal characteristics	Total (*N* = 5,484)	Housing type		Commercial facilities ^*^		Public transportation transit/stops ^*^		Sidewalks on streets ^*^		Shared paths for cycles and pedestrians ^*^		Free/low-cost recreational facilities ^*^	
		Apartment	House	Disagree	Agree	Disagree	Agree	Disagree	Agree	Disagree	Agree	Disagree	Agree
		4,552	932	1,221	4,263	672	4,812	1,436	4,048	1,916	2,568	1,281	4,203
Age (years)													
60–69	57.6 (3,157)	57.5 (2,618)	57.8 (539)	52.2 (638)	59.0 (2,519) ^△^	57.1 (384)	57.6 (2,773) ^△^	55.5 (797)	58.3 (2,360) ^△^	54.9 (1,052)	59.0 (2,105) ^△^	53.4 (684)	58.8 (2,473) ^△^
70–79	31.2 (1,711)	31.6 (1,438)	29.3 (273)	34.2 (417)	30.4 (1,294)	28.3 (190)	31.6 (1,521)	31.3 (449)	31.2 (1,262)	31.8 (609)	30.9 (1,102)	33.3 (426)	30.6 (1,285)
80+	11.2 (616)	10.9 (496)	12.9 (120)	13.6 (166)	10.6 (450)	14.6 (98)	10.8 (518)	13.2 (190)	10.5 (426)	13.3 (255)	10.1 (361)	13.3 (171)	10.6 (445)
Sex													
Male	49.0 (2,687)	48.8 (2,222)	49.9 (465)	49.1 (600)	49.0 (2,087)	51.2 (344)	48.7 (2,343)	48.7 (700)	49.1 (1,987)	48.1 (921)	49.5 (1,766)	47.6 (610)	49.4 (2,077)
Female	51.0 (3,797)	51.2 (2,330)	50.1 (467)	50.9 (621)	51.0 (2,176)	48.8 (328)	51.3 (2,469)	51.3 (736)	50.9 (2,061)	51.9 (995)	50.5 (1,802)	52.4 (671)	50.6 (2,126)
Residential area ^#^													
Urban	39.1 (2,144)	45.9 (2,089)	5.9 (55) ^△^	22.5 (275)	43.8 (1,869) ^△^	25.4 (171)	41.0 (1,973) ^△^	24.2 (347)	44.4 (1,797) ^△^	23.1 (443)	47.7 (1,701) ^△^	23.0 (295)	44.0 (1,849) ^△^
Suburban	60.9 (3,340)	54.1 (2,463)	94.1 (877)	77.5 (946)	56.2 (2,394)	74.6 (501)	59.0 (2,839)	75.8 (1,089)	55.6 (2,251)	76.9 (1,473)	52.3 (1,867)	77.0 (986)	56.0 (2,354)
Educational attainment (years of schooling)													
6-	49.6 (2,721)	45.2 (2,057)	71.2 (664) ^△^	62.4 (763)	45.9 (1,958) ^△^	56.7 (381)	48.6 (2,340) ^△^	63.3 (909)	44.8 (1,812) ^△^	64.0 (1,227)	41.9 (1,494) ^△^	61.0 (781)	46.2 (1,940) ^△^
7–12	45.0 (2,465)	48.4 (2,205)	27.9 (260)	33.3 (406)	48.3 (2,059)	40.0 (269)	45.6 (2,196)	33.2 (477)	49.1 (1,988)	32.9 (631)	51.4 (1,834)	35.6 (456)	47.8 (2,009)
13+	5.4 (298)	6.4 (290)	0.9 (8)	4.3 (52)	5.8 (246)	3.3 (22)	5.8 (276)	3.5 (50)	6.1 (248)	3.1 (58)	6.7 (240)	3.4 (44)	6.0 (254)
Marital status													
Single	13.8 (758)	12.9 (585)	18.6 (173) ^△^	16.5 (202)	13.0 (556) ^△^	17.0 (114)	13.4 (644) ^△^	17.0 (244)	12.7 (514) ^△^	16.8 (322)	12.2 (436) ^△^	15.3 (196)	13.4 (562)
Married/having a partner	86.2 (4,726)	87.1 (3,967)	81.4 (759)	83.5 (1,019)	87.0 (3,707)	83.0 (558)	86.6 (4,168)	83.0 (1,192)	87.3 (3,534)	83.2 (1,594)	87.8 (3,132)	84.7 (1,085)	86.6 (3,641)
Occupation ^†^													
White-collar	15.1 (827)	17.2 (781)	4.9 (46) ^△^	9.3 (114)	16.7 (713) ^△^	10.3 (69)	15.7 (758) ^△^	7.9 (114)	17.6 (713) ^△^	7.2 (137)	19.3 (690) ^△^	9.7 (124)	16.7 (703) ^△^
Salesperson	6.2 (338)	7.1 (321)	1.9 (17)	4.0 (49)	6.8 (289)	4.0 (27)	6.5 (311)	3.1 (44)	7.3 (294)	3.0 (58)	7.9 (280)	4.4 (56)	6.7 (282)
Blue-collar	78.7 (4,319)	75.7 (3,450)	93.2 (86.9)	86.7 (1,058)	76.5 (3,261)	85.7 (576)	77.8 (3,743)	89.0 (1,278)	75.1 (3,041)	89.8 (1,721)	72.8 (2,598)	85.9 (1,101)	76.6 (3,218)

§ PANES refers to the Physical Activity Neighborhood Environment Scale.

△ Differences in the items of the PANES by participants’ characteristics were statistically significant based on the chi-squared test.

* “Disagree” refers to “strongly or somewhat disagree,” while “Agree” refers to “strongly or somewhat agree” regarding the response to PANES-assessed neighborhood built environment attributes.

# Urban and suburban districts were defined based on the official classifications released by the China National Bureau of Statistics.

† Occupation referred to the job before retirement and was classified for each participant based on the recommendations by the China National Center for Chronic Non-communicable Disease Prevention and Control.

Table 3 presents the associations between perceived BE attributes and personal sociodemographic characteristics among community-dwelling older adults in Nanjing Municipality. After adjustment for potential confounding factors, age was associated only with housing type (item 1), showing that participants aged 70–79 years were less likely to report their housing type as a house compared to those aged 60–69 years. Interestingly, residential area was associated with all six BE attributes. Suburban participants were more likely to report their housing type as a house (OR = 10.07; 95% CI = 7.52, 13.50) but had lower odds of reporting PA-favorable commercial facilities (OR = 0.43; 95% CI = 0.36, 0.51), public transportation transit/stops (OR = 0.54; 95%CI = 0.44, 0.66), sidewalks on streets (OR = 0.55; 95%CI = 0.47, 0.64), shared paths for cycles and pedestrians (OR = 0.48; 95%CI = 0.42, 0.55), and free/low-cost recreational facilities (OR = 0.41; 95%CI = 0.34, 0.48). Moreover, participants with higher educational attainment were less likely to report their housing type as a house but were more likely to report PA-friendly commercial facilities (item 2), sidewalks on streets (item 4), shared paths for cycles and pedestrians (item 5), and free/low-cost recreational facilities (item 6). Compared to single individuals, those with a partner/spouse had lower odds of reporting their housing type as a house but were more likely to report PA-supportive sidewalks on streets (item 4) and shared paths for cycles and pedestrians (item 5). In addition, blue-collar workers were less likely to report PA-favorable sidewalks on streets (item 4) and shared paths for cycles and pedestrians (item 5). Notably, non-significant associations were examined between sex and each of the six BE attributes.

**Table 3:** Associations between personal characteristics and perceived built environment attributes among participants in this study (*N* = 5,484).

Personal characteristics	OR (95%CI) ^$^					
	Housing type (House vs. Apartment)	Commercial facilities ^*^ (Agree vs. Disagree)	Public transportation transit/stops ^*^ (Agree vs. Disagree)	Sidewalks on streets ^*^ (Agree vs. Disagree)	Shared paths for cycles and pedestrians ^*^ (Agree vs. Disagree)	Free/low-cost recreational facilities ^*^ (Agree vs. Disagree)
Age (years)						
60–69	1	1	1	1	1	1
70–79	0.77 (0.65, 0.92) ^‡^	0.89 (0.77, 1.03)	1.20 (0.99, 1.46)	1.08 (0.93, 1.24)	1.03 (0.90, 1.18)	0.94 (0.81, 1.09)
80+	0.91 (0.70, 1.17)	0.84 (0.67, 1.05)	0.89 (0.69, 1.16)	0.95 (0.77, 1.17)	0.89 (0.73, 1.09)	0.87 (0.70, 1.08)
Sex						
Male	1	1	1	1	1	1
Female	0.81 (0.69, 1.00)	1.12 (0.98, 1.29)	1.17 (0.98, 1.39)	1.12 (0.98, 1.28)	1.10 (0.98, 1.25)	1.01 (0.89, 1.16)
Residential area ^#^						
Urban	1	1	1	1	1	1
Suburban	10.07 (7.52, 13.50) ^‡^	0.43 (0.36, 0.51) ^‡^	0.54 (0.44, 0.66) ^‡^	0.55 (0.47, 0.64) ^‡^	0.48 (0.42, 0.55) ^‡^	0.41 (0.34, 0.48) ^‡^
Educational attainment (years of schooling)						
6-	1	1	1	1	1	1
7–12	0.64 (0.53, 0.76) ^‡^	1.34 (1.14, 1.57) ^‡^	1.02 (0.84, 1.24)	1.41 (1.21, 1.64) ^‡^	1.50 (1.30, 1.72) ^‡^	1.24 (1.06, 1.45) ^‡^
13+	0.28 (0.13, 0.59) ^‡^	0.93 (0.65, 1.33)	1.25 (0.76, 2.04)	1.18 (0.83, 1.69)	1.41 (1.01, 1.97) ^‡^	1.26 (0.87, 1.82)
Marital status						
Single	1	1	1	1	1	1
Married/having a partner	0.75 (0.61, 0.92) ^‡^	1.09 (0.93, 1.32)	1.16 (0.92, 1.47)	1.20 (1.01, 1.44) ^‡^	1.21 (1.02, 1.44) ^‡^	0.96 (0.79, 1.16)
Occupation ^†^						
White-collar	1	1	1	1	1	1
Salesperson	0.63 (0.34, 1.15)	0.96 (0.66, 1.40)	1.16 (0.72, 1.86)	1.20 (0.82, 1.77)	1.09 (0.77, 1.56)	0.92 (0.64, 1.31)
Blue-collar	1.32 (0.93, 1.87)	0.81 (0.64, 1.04)	0.85 (0.64, 1.15)	0.63 (0.50, 0.80) ^‡^	0.54 (0.44, 0.68) ^‡^	0.87 (0.69, 1.10)

$ OR: odds ratio; CI: confidence interval. They were calculated using mixed-effects multivariate logistic regression models, with mutual adjustment for age, sex, residential area, educational attainment, marital status, occupation, physical activity, sedentary behavior, body weight status, diabetes, hypertension, stroke, coronary heart disease, chronic kidney disease, chronic pulmonary disease, cancer, sleep quality, depressive symptoms, frailty, and sampling community as a random effect. In the analyses, “Apartment” and “Disagree” were used as the reference groups, respectively.

* “Disagree” refers to “strongly or somewhat disagree,” while “Agree” refers to “strongly or somewhat agree” regarding the response to PANES-assessed neighborhood built environment attributes.

# Urban and suburban districts were defined based on the official classifications released by the China National Bureau of Statistics.

† Occupation referred to the job before retirement and was classified for each participant based on the recommendations by the China National Center for Chronic Non-communicable Disease Prevention and Control.

‡ Statistically significant.

## 4 Discussion

This population-based study aimed to investigate the associations between perceived neighborhood BE attributes and the six typical sociodemographic characteristics among community-dwelling adults aged 60 years and older in Nanjing, China. It was observed that selected perceived PA-related BE attributes (core items 1–6 of the PANES) were associated with five of the six common sociodemographic characteristics (except sex) in the study.

Previously, three studies investigated the relationship between walking and perceived BE attributes. They also reported the distribution of perceived BE attributes according to selected sociodemographic characteristics as secondary outcomes (21–23). Of these three studies, the first two were conducted in Shanghai and Hong Kong, China, while the third was conducted in Seoul, Korea (21–23). The Shanghai and Hong Kong studies used the Chinese version of the Abbreviated Neighborhood Environment Walkability Scale (ANEWS) to measure perceived BE attributes, while the Korean study used the Korean version of the PANES (21–23). Notably, as for the outcome measure, all three studies used an integrated index computed based on perceived BE attributes rather than analyzing each individual BE attribute item separately, similar to the approach employed in the current study (21–23). Among the 1,528 participants aged 15–75 years in the Shanghai study, significant differences in perceived environmental attributes were observed by occupation but not by age or sex (21). For the 181 participants aged 60 years or above in the Hong Kong study, age, sex, educational level, and occupation were not associated with BE attributes, while married individuals reported higher levels of walkability compared to their single counterparts (22). Among the 2000 participants aged 18–69 years in the Seoul study, only sex was not associated with the PANES score (23). Overall, the associations between sociodemographic characteristics and BE attributes were inconsistent across these three existing studies (21–23).

As participants and outcome measures differed between these three studies and the present one, their findings are not directly comparable with ours (21–23). Moreover, no evidence-based mechanisms are available for explaining the relationship between sociodemographic characteristics and perceived BE attributes, as little is known about the specific factors that influence residents’ ability to accurately perceive BE attributes (47). This is a big challenge for us attempting to make direct explanations for the findings. Therefore, we have to try to explain the associations between sociodemographic characteristics and perceived BE attributes mainly based on the intrinsic evidence found in the current study.

Compared to their urban counterparts, suburban residents were more likely to report living in houses (item 1), which indicates lower neighborhood residential density in the PANES, but were less likely to perceive the other five PA-favorable BE attributes (item 2–6). These findings might be explained by the fact that suburban areas typically have lower population density and fewer public transport transit/stops, commercial and recreational facilities, street sidewalks, and paths for cycles and pedestrians compared to urban areas in China. Participants with higher educational attainment were less likely to report their housing type as a house relative to those who were less educated. This might be due to the fact that only a small proportion of less-educated residents lived in urban areas (19.1%) among the participants in this study. Conversely, older residents with 7–12 years of education, but not those with 13 + years, were more likely to report access to PA-friendly commercial/recreational facilities, sidewalks on streets, and shared paths for cycles and pedestrians compared to their counterparts with 0–6 years of education. This might be partially explained by the fact that very few older residents (5.4% only) had 13 + years of schooling in this study. Participants who had a spouse/partner had lower odds of reporting their housing type as a house but higher odds of reporting sidewalks on streets and shared paths for cycles and pedestrians in their neighborhoods relative to single persons. This might be attributable to the fact that the majority of single residents (67.3%) in this study lived in suburban areas. Blue-collar workers were less likely to report the presence of sidewalks on streets or shared paths for cycles and pedestrians compared to white-collar workers. This might be explained by the fact that blue-collar workers are usually less educated (48).

Recently, neighborhood BE attributes have been examined as potential determinants of PA and related chronic conditions among residents, particularly older individuals (2–4, 49–51). Moreover, perceived BE features may influence residents’ PA and health state (52). Notably, a recent series of articles published in The Lancet highlighted that science-based city planning can provide effective solutions to some key health-related behaviors (e.g., PA) and chronic conditions at the community level (2–4). China has experienced rapid urbanization alongside sustained economic growth over the past several decades (53). Meanwhile, China has also witnessed a rapid increase in its older population, with 264 million (18.7% of the total population) adults aged 60 years and older in 2020 (54). Therefore, for developing BE-based PA interventions in China, it is of public health importance to identify BE attributes that are sensitive to residents’ sociodemographic characteristics.

Regarding the agreement between subjectively perceived and objectively measured BE attributes, no strong agreement between them has been reported among participants in the context of Western communities (13, 55–57). However, the agreement between subjectively perceived and objectively measured PANES core items was recently examined among adults in China, revealing acceptable agreement between them (30). This suggests that the perception of PANES-based BE attributes may be population-sensitive, highlighting that sociodemographic characteristics may play a role in perceiving and potentially utilizing a PA-friendly BE.

Moreover, according to the KAP theory, if individuals do not perceive or recognize their neighborhood BE as PA-friendly or walkable, they are unlikely to be aware of the actual PA-supportive BE attributes available to them (8). Consequently, they may be less likely to utilize these PA-favorable neighborhood BE attributes for engaging in PA (e.g., walking). Therefore, it is reasonable to suggest that subjectively perceived neighborhood BE attributes may be more meaningful and important than objectively measured attributes in enabling local residents to intentionally engage in PA. Furthermore, from the public health perspective, it is plausible that elevating awareness of PA-friendly BE attributes among residents may be a potentially effective strategy for population-based PA-promotion interventions.

This study has several strengths. First, participants were randomly selected from the entire municipality of Nanjing and were representative of the overall 1.9 million local older adults (34). Second, the instrument used to assess perceived BE attributes was the validated Chinese version of the PANES, which has demonstrated good validity and reliability against objectively measured BE attributes among adults in China (29, 31). Third, all six classical personalized sociodemographic characteristics were analyzed. Fourth, consistent findings were observed regarding the relationship between individual BE attributes and sociodemographic characteristics. Finally, this study has significant public health implications, suggesting that sociodemographic characteristics may be used as helpful markers for screening local residents who may perceive PA-friendly BE attributes in population-level PA-promotion campaigns.

The study limitations should also be acknowledged. First, the cross-sectional nature of the study did not allow us to infer any causality between personal sociodemographic characteristics and neighborhood BE attributes. Second, sociodemographic characteristics were self-reported, implying potential recall bias. Third, information on lifestyle, behaviors, and chronic conditions was also self-reported and may therefore be subject to underreporting or misclassification. Fourth, in this study, multiple tests were conducted to examine the associations between sociodemographic characteristics and six perceived built environment attributes. This may potentially increase the possibility of significant findings by chance. Therefore, it should be emphasized that the analyses were exploratory and that the statistically significant findings should be interpreted cautiously. Fifth, the original 4-point Likert-scale responses to all PANES items were dichotomized for analysis to ensure comparability with previous studies. However, it is clear that this data-classification approach may reduce variability and result in a loss of information. Sixth, the available PA-associated factors were controlled for in the analysis, which may imply potential overadjustment of the statistical effect size. Seventh, the mechanisms underlying the associations between sociodemographic characteristics and BE attributes are difficult to establish (47). Consequently, the relationship was mainly explained based on the internal evidence from the present study itself. Lastly, only objectively measurable BE attributes corresponding to the PANES core items were analyzed; therefore, other potentially relevant aspects of the neighborhood BE might not have been fully captured (10).

In conclusion, residential area, educational attainment, occupation, and marital status were each associated with perceived BE attributes among older residents in regional China, while age was only associated with housing type. In the context of rapid urbanization and population aging in China, this study has significant public health implications, suggesting that sociodemographic characteristics may serve as helpful markers for identifying older adults who are sensitive to specific PA-favorable built environment attributes in population-level PA-promotion campaigns in regional China.

## Data Availability

The original contributions presented in the study are included in the article/supplementary material, further inquiries can be directed to the corresponding authors.
